# Occurrence and Molecular Characterization of Alphaherpesvirus in Psittacines in the State of Mato Grosso

**DOI:** 10.1155/vmi/6417751

**Published:** 2026-08-23

**Authors:** Thais Rosso da Silva, Marco Túlio dos Santos Costa, Danny Franciele da Silva Dias Moraes, Camila Regina Almeida Vani, Augusto Cesar dos Santos Stering, Valéria Dutra, Luciano Nakazato

**Affiliations:** ^1^ Department of Microbiology and Molecular Biology, College of Veterinary Medicine, Federal University of Mato Grosso, Av. Fernando Corrêa da Costa 2367 Boa Esperança, Cuiabá, Mato Grosso, Brazil, ufmt.br

**Keywords:** animal health, birds, herpesvirus, molecular diagnosis, Pacheco’s disease, spillover

## Abstract

The Alphaherpesvirinae subfamily belongs to the Orthoherpesviridae family, whose viruses are characterized by rapid replication and the ability to establish latent infection in host cells. These viruses cause important avian diseases such as Marek’s disease, infectious laryngotracheitis, and Pacheco’s disease (PD) in psittacines. In Brazilian avifauna, especially within the Psittacidae family, sanitary problems are frequently observed due to stress caused by deforestation and wildlife trafficking, factors that can exacerbate herpesvirus infections. Due to the limited available data on herpesvirus occurrence in wild psittacines, the aim of this study was to survey and identify alphaherpesviruses in psittacine species in Mato Grosso using the polymerase chain reaction (PCR) technique, correlating the results with phylogenetic analysis. Biological samples were obtained from IBAMA, SEMA, and CEMPAS and sent to the Veterinary Hospital of UFMT (HOVET) between 2019 and 2024. PCR‐positive samples, which amplified a 264‐bp fragment of the UL30 gene, were subjected to sequencing and phylogenetic analysis. In total, 131 samples were analyzed, including liver tissue, cloacal swabs, and blood, from both captive and free‐living birds. Twelve (9.16%) were positive by PCR, with swabs showing the highest detection rate. A sequenced sample from *Amazona aestiva* showed 79.77% amino acid identity with *Eupsittula herpesvirus*, suggesting a potentially distinct alphaherpesvirus lineage. Another positive sample from *Ara ararauna* showed 97.65% identity with *Meleagrid alphaherpesvirus 1*. These results demonstrate the detection of alphaherpesviruses in psittacine birds from Mato Grosso and reveal phylogenetic relationships that warrant further genomic investigation to better understand viral diversity and potential host associations.

## 1. Introduction

The Alphaherpesvirinae subfamily belongs to the Orthoherpesviridae family, which also includes the Betaherpesvirinae and Gammaherpesvirinae subfamilies. The Alphaherpesvirinae subfamily comprises five genera (Iltovirus, Mardivirus, Scutavirus, Simplexvirus, and Varicellovirus), whose members are characterized by their ability to infect a wide range of vertebrate hosts [1]. They are large, enveloped DNA viruses that establish lifelong latent infections in their natural hosts as a result of long‐term coevolution. These viruses cause several diseases in birds, such as infectious laryngotracheitis, Marek’s disease, and Pacheco’s disease (PD). Alphaherpesviruses are typically transmitted through direct contact with infected secretions or aerosols and may reactivate under immunosuppressive conditions, causing severe disease, particularly following transmission between different species [2].

When focusing on the avian family Psittacidae, *Psittacid alphaherpesvirus 1* (PsHV‐1) is highly relevant due to the susceptibility of these birds to infection by this agent [2]. PD, caused by PsHV‐1, can lead to a severe systemic disease that often results in acute hepatic necrosis and sudden death [3, 4]. PD was first identified in 1930, and since then, outbreaks with high mortality in psittacines around the world have been related to its etiological agent [5]. Despite its importance, the specific knowledge regarding herpesvirus infection, genetic diversity, and epidemiology in neotropical psittacines remains limited.

According to the International Union for Conservation of Nature (IUCN) [6], 113 psittacine species worldwide are classified as critically endangered, endangered, or vulnerable. In Brazil, which hosts a vast diversity of psittacines, many species face significant threats. This scenario is attributed to factors such as deforestation, habitat fragmentation, agricultural activities, urban expansion, fires, and, especially, the illegal capture of animals for trade [7]. Environmental stress and overcrowding in captivity or during trafficking can increase susceptibility to viral infection or reactivation [8]. Understanding viral circulation is crucial for conservation planning, as herpesvirus outbreaks could contribute to population declines and pose risks to both captive and free‐living populations.

Due to the limited knowledge regarding herpesvirus circulation in wild and captive psittacines in Brazil, the aim of this study was to survey and identify alphaherpesviruses in psittacine species in the state of Mato Grosso, contributing to the understanding of viral diversity in these populations.

## 2. Material and Methods

### 2.1. Sample Collection

The collection and handling of biological samples for this study followed the guidelines approved by the Animal Ethics Committee (CEUA), in accordance with protocol *n*° 23,108.064286/2021‐91. The stored samples are part of a previous study for the analysis of other avian pathogens [9] according to CEUA protocol *n*° 23,108.053296/2020‐11.

Biological samples of blood, liver, and cloacal swabs were collected from wild birds and sent to the Veterinary Hospital of the Federal University of Mato Grosso (HOVET) by SEMA (State Secretariat for the Environment of Mato Grosso) and IBAMA (Brazilian Institute of the Environment and Renewable Natural Resources). In addition, samples from birds kept in captivity at the Center for Medicine and Research on Wild Animals of the Federal University of Mato Grosso (CEMPAS‐UFMT) were included.

The study included 131 samples belonging to the order Psittaciformes, distributed as follows: *Ara macao* (scarlet macaw; *n* = 2), *Ara ararauna* (blue‐and‐yellow macaw; *n* = 116), *Ara chloropterus* (red‐and‐green macaw; *n* = 3), *Primolius maracana* (blue‐winged macaw; *n* = 1), and *Amazona aestiva* (turquoise‐fronted amazon; *n* = 9). Most of the samples consisted of materials obtained from captive animals, accounting for 98.47% of the total.

The samples were collected between 2019 and 2024 at HOVET. Blood and swab samples were collected from live animals during routine clinical examinations or quarantine screening. Liver samples were obtained during necropsies of animals that had died, for epidemiological investigation purposes. Subsequently, the samples were sent to the Molecular Biology Laboratory, where they were properly stored at −20°C until DNA extraction and subsequent molecular analyses.

### 2.2. DNA Extraction

DNA extraction was performed using the MagMAX kit (Thermo Fisher), according to the manufacturer’s instructions. For the whole blood samples, 100 µL stored in a 1.5‐mL microtube was used; for liver samples, 20 mg; and for swab samples, 150 µL of the saline solution in which the material was submerged to release the genetic material. After extraction, the DNA was eluted in 70 µL of elution buffer (10‐mM Tris and 0.1‐mM EDTA), and the material was stored at −20°C.

### 2.3. Polymerase Chain Reaction (PCR)

The extracted samples were subjected to conventional PCR (cPCR) for the detection of Alphaherpesvirus, amplifying a 264‐base pair (bp) fragment in the DNA polymerase (UL30) gene region, according to a modified protocol based on Okoh et al. (2023) [4], using the following oligonucleotide primers: F 5′‐AGCATHATYCAGGCBCAYAAYCTSTGYTTYA and R 3′‐TTRATBGCVRVCTGYTGYTTRTC.

For the PCR reaction, 2 μL of DNA in 18 μL were used in the mix composed of 1X Buffer 10x (Sigma), 2.5‐mM MgCl2 (Sigma), 0.2‐mM dNTPs, 1U JumpStart RED Taq DNA Polymerase, and 0.4 μM of each primer and sterile ultrapure water in adequate quantity until reaching the final volume of 20 μL. *Gallid alphaherpesvirus 2* (GaHV‐2) DNA was used as a positive control, and ultrapure water was used as a negative control. The Touchdown protocol used in the ProFlex PCR System thermocycler is shown in Table 1.

**TABLE 1:** Touchdown protocol in the thermocycler for the diagnosis of alphaherpesvirus.

	Temperature	Time
1. Initial denaturation	95°C	5 minutes
10 cycles of:		
2. Denaturation	94°C	1 minute
3. Annealing	58°C (−1°C/cycle)	1 minute
4. Extension	72°C	2 minutes
35 cycles of:		
5. Denaturation	94°C	1 minute
6. Annealing	48°C	1 minute
7. Extension	72°C	2 minutes
1 cycle of:		
8. Final extension	72°C	10 minutes
9. ∞	4°C	infinity

The results of the PCR reactions were visualized after fractionation on a 1.5% agarose gel stained with GelRed Nucleic Acid Gel Stain (Biotium) and documented on the ChemiDoc XRS system (Bio‐Rad).

### 2.4. Sequencing and Phylogenetic Analysis

For sequencing, the PCR‐positive samples were purified with the Ampure XP kit and the products were sequenced by the Sanger method [10]. The sequences were analyzed using an automatic capillary ABI 3500 DNA Analyzer, resulting in a sequence for each primer of all the samples sent. The sequence data were analyzed and their quality checked using the CLC DNA Workbench 6.0 software (*Q* > 20). Both forward and reverse reads were analyzed to generate a consensus sequence prior to phylogenetic analysis.

The sequenced samples were compared using the BLAST tool (NCBI) with the protein gene sequences already deposited in GenBank. Reference sequences representing the currently recognized species of the genera Mardivirus and Iltovirus were retrieved from GenBank according to ICTV taxonomy (https://ictv.global/taxonomy). The reference dataset included multiple PsHV‐1 sequences, GaHV‐2, infectious laryngotracheitis virus (ILTV), and other representative avian alphaherpesvirus sequences. Sequences were aligned using MAFF, and low‐quality regions were trimmed using BMGE. Model selection was performed using the Akaike information criterion (AIC), which identified the GTR + I + G model as the best‐fit evolutionary model. The phylogenetic tree was reconstructed using maximum likelihood (ML) inference implemented in PhyML with 1000 bootstrap replicates to assess clade support.

## 3. Results

Of the 131 total samples, 12 (9.16%) showed amplification of PCR products with the expected size. Information regarding the clinical characteristics of the sampled animals was limited, and most did not present clinical signs at the time of sampling. Regarding the sampling site, cloacal swabs were the material with the highest number of positive samples (*n* = 9), followed by liver samples (*n* = 2). Blood samples showed only one positive result. The distribution of infection among the different species is shown in Table 2.

**TABLE 2:** Detection of Alphaherpesvirinae by PCR in Psittacidae in the state of Mato Grosso.

Species (common name)	*N*	Habitat	Swabs (+)	Liver (+)	Blood (+)
*Amazona aestiva* (turquoise‐fronted amazon)	9	Captive	3	0	0
*Ara ararauna* (blue‐and‐yellow macaw)	116	Captive	6	2	1
*Ara chloropterus* (red‐and‐green macaw)	3	Free living	0	0	0
*Ara macao* (scarlet macaw)	2	Captive	0	0	0
*Primolius maracana* (blue‐winged macaw)	1	Captive	0	0	0
Total	131				

*Note: N*: Total number of birds sampled. (+) Number of positive samples by PCR. For *Ara ararauna*, each positive result (swab, liver, or blood) originated from a distinct individual, totaling 9 positive birds for this species.

All 12 PCR‐positive samples were submitted for Sanger sequencing. However, 10 samples were unsuccessful in yielding readable sequences, likely due to low concentrations of purified viral DNA. Two swab samples were successfully sequenced and confirmed as alphaherpesviruses: one from *A. aestiva* and another from *A. ararauna*, both from adult individuals with no clinical history.

**FIGURE 1:**
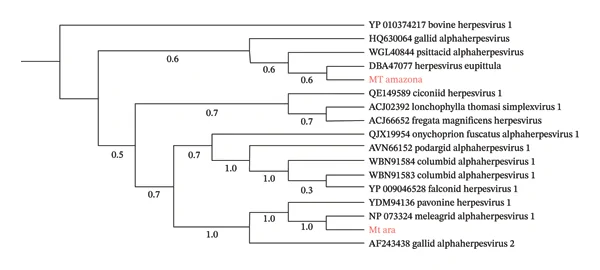
The phylogenetic tree was constructed with MAFF alignment of amino acids. Model selection was performed using the Akaike information criterion (AIC), which identified the GTR + I + G model as the best‐fit evolutionary model. The phylogenetic tree was reconstructed using maximum likelihood (ML) inference implemented in PhyML with 1000 bootstrap replicates to assess clade support.

The phylogenetic tree of amino acid sequences of the DNA polymerase (UL30) gene, presented in Figure 1, shows the relationship of the samples detected in this study. The phylogenetic analysis demonstrates the clustering of the samples from this study with avian alphaherpesviruses. The MT Amazona sample, corresponding to the sequence detected in *A. aestiva*, formed a distinct clade, sharing 79.77% amino acid identity with *Eupsittula herpesvirus* (DBA47077). The MT Amazona gene sequence obtained in this study was deposited in GenBank under accession number PQ650704. The analysis, with a bootstrap value of 0.946 (94.6%), indicates a considerable level of confidence in this clade. In addition, the comparison between the MT Ara sample revealed a significant proximity to the *Meleagrid alphaherpesvirus 1* (NP073324) sequence in the database, showing a genomic similarity of 97.65% and forming a clade with a bootstrap value of 0.999 (99.9%).

## 4. Discussion

The detection rate of alphaherpesviruses varies considerably among studies. A study conducted by the Federal University of Minas Gerais found approximately 60% of the diagnostic samples positive for PsHV [11]. However, this result contrasts with the data obtained in the present study, which identified a frequency of approximately 9% positive samples. This comparison should be interpreted with caution, as methodological, design, and population differences between studies limit the direct comparability of the findings. The high prevalence in diagnostic submissions often reflects biased sampling toward sick birds, whereas our study included routine screening.

Among the psittacine species analyzed, only two showed positive PCR results. In *A. aestiva*, 3 out of 9 swabs (33.3%) were positive. In *Ara ararauna*, which had the largest number of samples, 6 out of 116 swabs (5.2%) tested positive, in addition to 2 liver samples (1.7%) and 1 blood sample (0.9%). These findings highlight the importance of considering the type of sample when interpreting prevalence.

Cloacal swab samples showed the highest number of positive detections, which is consistent with the findings of Žlabravec et al. [12]. This high detection rate likely reflects viral shedding from epithelial tissues of the respiratory and digestive tracts, which are primary sites of alphaherpesvirus replication. Alphaherpesviruses are known to undergo primary replication in epithelial tissues, and the cloacal region serves as a convergence point for secretions from both systems, facilitating viral detection in swab samples. While these findings do not necessarily demonstrate active intestinal replication, they are consistent with the known pathobiology of herpesvirus infections in birds, where epithelial shedding is a characteristic feature of infection [13, 14].

To our knowledge, this is the first report of an alphaherpesvirus detection in *A. aestiva* based on molecular characterization. The positive sample from *A. aestiva* showed 79.77% amino acid similarity to the *Eupsittula herpesvirus* sequence, which was obtained from the feather shaft pulp of *Eupsittula canicularis* in the United States (USA), based on the short UL30 fragment. This relatively low percentage of identity suggests that this may represent a distinct alphaherpesvirus lineage rather than the same species. The novelty of this finding in *A. aestiva* is significant for understanding alphaherpesvirus diversity in neotropical psittacines. While cross‐species transmission (spillover) is a possibility, herpesviruses generally exhibit a high degree of host specificity. The genetic divergence from *Eupsittula herpesvirus* and the detection in *A. aestiva* raise the possibility that this virus represents a host‐specific alphaherpesvirus lineage; however, the limited sample size and low overall prevalence do not provide sufficient evidence to definitively establish this conclusion at present. This remains a hypothesis that warrants further investigation through expanded sampling of *A. aestiva* populations and complete genomic sequencing to clarify the host–virus relationships and evolutionary history of this lineage.

The swab sample from *Ara ararauna* showed a similarity of 97.65% with *Meleagrid alphaherpesvirus 1*, known as turkey herpesvirus and widely used as a vaccine strain for Marek’s disease [15]. Given that the samples originated from captive animals, this high similarity warrants consideration of environmental exposure or cross‐species transmission in a captive setting, possibly related to proximity to vaccinated poultry or contaminated environments.

The study has limitations, including the small number of successfully sequenced samples and the predominance of captive birds. The low sequencing success rate for PCR‐positive samples highlights the challenges of obtaining sufficient viral DNA from subclinical shedding. Future studies with broader genomic sequencing and larger sample sizes of free‐living populations are necessary to fully elucidate the diversity and epidemiology of alphaherpesviruses in neotropical psittacines.

## 5. Conclusion

This study identified the presence of alphaherpesviruses in captive psittacine birds in Mato Grosso. Sequence analysis of a partial UL30 gene fragment revealed a lineage phylogenetically related to *Eupsittula herpesvirus* in *A. aestiva* and a sequence highly similar to *Meleagrid alphaherpesvirus 1* in *Ara ararauna*. These findings provide additional molecular evidence of distinct alphaherpesvirus lineages in psittacine birds from Mato Grosso. Due to the nature of herpesvirus infections, molecular detection methods such as PCR are relevant tools for health monitoring. Further comprehensive genomic studies are needed to better understand viral diversity, host specificity, and the implications for the conservation and management of psittacine species.

## Funding

This study was funded by Coordenação de Aperfeiçoamento de Pessoal de Nível Superior, 155/2021.

## Conflicts of Interest

The authors declare no conflicts of interest.

## Data Availability

The data that support the findings of this study are available from the corresponding author upon reasonable request.
